# Association of choroid plexus volume with white matter microstructure, glymphatic function, and peripheral systemic inflammation in Alzheimer’s disease

**DOI:** 10.1038/s41398-025-03432-1

**Published:** 2025-07-11

**Authors:** Huwei Xia, Yijia Feng, Han Zhu, Danlu Yang, Chaoqun Wang, Zhipeng Wang, Hanyuan Zhang, Wenhao Pan, Yifan Zhao, for the Alzheimer’s Disease Neuroimaging Initiative, Weihong Song, Yili Wu

**Affiliations:** 1 Center for Geriatric Medicine, Zhejiang Key Laboratory of Alzheimer’s Disease, Oujiang Laboratory (Zhejiang Lab for Regenerative Medicine, Vision and Brain Health), Zhejiang Provincial Clinical Research Center for Mental Health, Institute of Aging, Wenzhou Medical University, Wenzhou, Zhejiang 325000 China; 2 Wenzhou Key Laboratory of Basic and Translational Research for Mental disorders, Zhejiang Provincial Clinical Research Center for Mental Health, Key Laboratory of Alzheimer’s Disease of Zhejiang Province, School of Mental Health and The Affiliated Wenzhou Kangning Hospital, Institute of Aging, Oujiang Laboratory (Zhejiang Lab for Regenerative Medicine, Vision and Brain Health), Wenzhou Medical University, Wenzhou, Zhejiang 325000 China

**Keywords:** Diseases, Predictive markers

## Abstract

There has been growing attention to the role of choroid plexus (CP) in neurodegenerative diseases. However, its relationship with various pathophysiological changes in Alzheimer’s Disease (AD) remains unclear. The purpose of this study is to investigate the relationship between CP volume (CPV) and white matter microstructure, cognitive function, glymphatic function, and peripheral systemic inflammation in AD. A total of 1351 participants with cognitive impairment who had available 3 T MRI scans were included from ADNI. CPV was automatically segmented using Gaussian Mixture Model (GMM). The Mini-Mental State Examination (MMSE) was employed to assess cognitive function. PSMD and DTI-ALPS based on DTI sequence were used to reflect white matter microstructure and glymphatic system. Peripheral systemic inflammation was represented by the neutrophil-lymphocyte ratio (NLR). Group comparisons and correlations were adjusted for age, sex, education and APOE4 carrier status. Participants with AD exhibited larger CPV (*p* < 0.001), higher PSMD (*p* < 0.001) and NLR (*p* = 0.035), and lower DTI-ALPS (*p* < 0.001) compared to those with subjective cognitive decline (SCD). CP enlargement was independently associated with higher PSMD (β = 0.223, *p* < 0.001) and worse cognitive function both cross-sectionally (β = −0.212, *p* < 0.001) and longitudinally (β = −0.214, *p* < 0.001). Furthermore, PSMD partially mediates the impact of CP enlargement on the severity and progression of cognitive function. Partial correlation analysis revealed that CP enlargement was associated with higher NLR (r = 0.101, *p* = 0.001) and lower DTI-ALPS (r = −0.241, *p* < 0.001). These findings suggest that CPV may reflect underlying pathophysiological processes in AD and serve as a biomarker for white matter damage and cognitive impairment progression.

## Introduction

Alzheimer’s disease (AD) is one of the most common neurodegenerative diseases that is characterized by progressive memory loss and cognitive impairment [1]. It is estimated that 6.7 million Americans age 65 and older are currently afflicted with AD. This number will more than double and could reach 13.8 million by 2060, assuming no cure or effective treatment for slowing down the disease progression. The main pathological changes of AD are the accumulation of β-amyloid (Aβ) plaques and hyperphosphorylated Tau [2–6]. However, the true etiology of AD remains unknown, and various pathophysiological alterations may jointly contribute to its formation such as white matter microstructural changes, glymphatic dysfunction, and peripheral inflammation activation [7–10].

The choroid plexus (CP) is a veil-like structure containing double-layered epidermal cells, blood vessels, fibroblasts, and immune cells located in brain ventricles [11]. Functionally, the CP is responsible for secreting almost 60–80% of cerebrospinal fluid (CSF) and forms the blood-cerebrospinal fluid barrier, which involves in the maintenance of a homeostatic environment within the central nervous system (CNS) [12]. Due to its unique interface between the peripheral blood and the brain, and the highly fenestrated nature of the capillary endothelium, it serves as a gateway for immune cells and specific molecules trafficking into the brain [13]. The CP has also been considered as a regulator impacting leukocyte migration, inflammatory responses, cognitive function, circadian rhythms, and the gut-brain axis [12]. Although the importance of CP in CNS was reported long ago [14], it has remained one of the least explored CNS structures in clinical research. Only recently has it attracted attention of neuroscientists as a potential biomarker for the diagnosis, prognosis, and treatment of neurodegenerative disorders.

Postmortem autopsy confirmed several morphological changes in the CP of AD patients, including epithelial atrophy and basement membrane thickening [15]. Additionally, CP transcriptomic studies detected alterations in immune-related pathways of AD patients [16]. These studies highlight an important role for the CP in the AD pathophysiological process. However, there are only a few in vivo imaging studies on it. A recent study revealed that CP enlargement is associated with the severity of cognitive impairment in the AD continuum [17]. Another study found that enlarged CP volume (CPV) was linked to increased Aβ deposition and memory deterioration [18]. Accumulating evidence indicated that white matter damage is a common phenomenon among neurodegenerative diseases, with the onset of these disorders aligning with notable changes in myelin [19]. This implies that alterations in white matter function could be a principal factor driving dysfunction. Recent studies further indicated that white matter regions are particularly vulnerable to aging and neurodegenerative processes compared to grey matter regions, highlighting the accelerated aging of glial cells within white matter areas, which may significantly contribute to functional decline [20]. Moreover, PET imaging revealed that Aβ accumulation correlates with reduced myelin content in several brain regions, further supporting the idea that white matter injury is the earliest detectable abnormalities prior to the clinical symptoms of AD [21]. Taken together, the CP enlargement and white matter changes associated with AD have not been clarified. The hypothesis in this study is that in patients with enlarged CPV, there is a potential for exacerbated and accelerated white matter injury, consequently leading to a more rapid decline in cognitive function. Additionally, the CP, serving as an important site for immune communication between the periphery and the brain, as well as the site of CSF production, may reflect the activation of systemic inflammation and impaired glymphatic function in AD. Therefore, our main objective is to: (1) investigate the relationship between CPV and white matter microstructure and their impact on cognition, both cross-sectionally and longitudinally. Further explore the potential role of white matter microstructure as a mediator in the CPV-cognition relationship; the secondary objectives are to (2) assess the links between CPV and glymphatic function; and (3) evaluate the correlation between CPV and peripheral systemic inflammation.

## Materials and methods

### Study participants

Participants included in this study were from Alzheimer’s Disease Neuroimaging Initiative (ADNI) database (https://adni.loni.usc.edu/). The ADNI is a pioneering collaborative effort in Alzheimer’s research, focusing on serial MRI, PET, and diverse biomarkers, along with robust clinical assessments. ADNI seeks to validate measures for the early diagnosis of late-onset AD and track the progression of mild cognitive impairment (MCI). With ethical approval and participant consent, ADNI stands as a crucial public database, providing support for research, facilitating treatment development, and streamlining Alzheimer’s clinical trials. This multidisciplinary initiative holds substantial promise for advancing the diagnosis and intervention of AD.

**Fig. 1:**
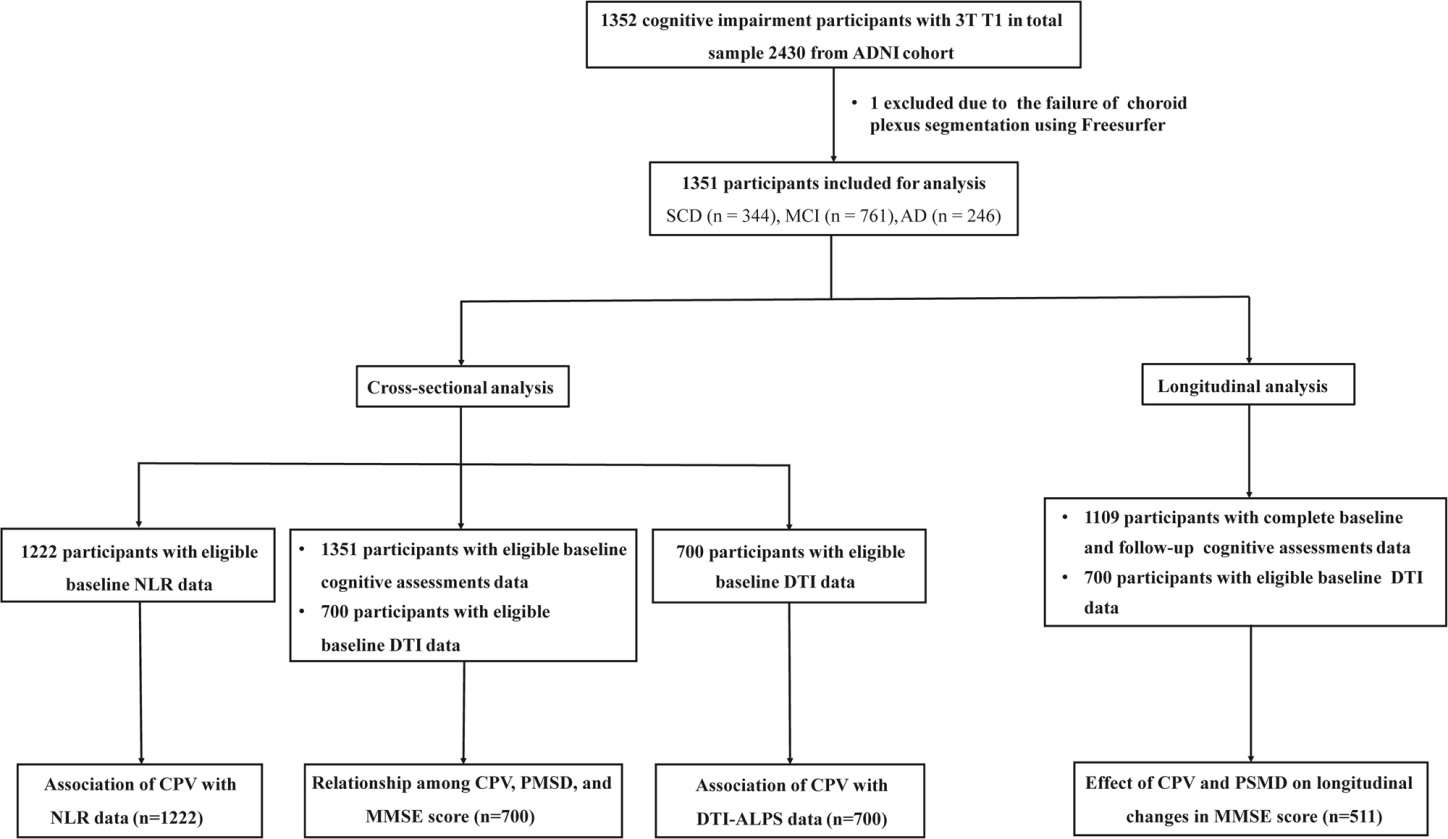
Flowchart of participants selection. SCD subjective cognitive decline, MCI mild cognitive impairment, AD Alzheimer’s disease, CPV choroid plexus volume, NLR neutrophil-lymphocyte ratio, DTI-ALPS DTI-analysis along the perivascular space, PSMD peak width of skeletonized mean diffusivity, MMSE Mini-Mental State Examination.

We identified 1352 participants with cognitive impairment from ADNI, all of whom had baseline 3 T MRI scans. The image quality of these scans was carefully controlled. First, the scans were automatically checked for protocol compliance, then manually reviewed by trained analysts to assess artifacts and anatomical coverage. As a result, only one participant’s scan failed during FreeSurfer processing. Ultimately, 1351 participants were included in our analysis. All participants had their baseline visit between 2006 and 2023. Inclusion criteria were (1) available baseline 3 T MRI scans, with successful segmentation of the CPV achievable through Freesurfer 7.3. (2) a baseline diagnosis of subjective cognitive decline (SCD, *n* = 344), MCI (n = 761), or AD (*n* = 246). Among them, 1222 participants had available neutrophil-lymphocyte ratio (NLR), and 700 participants had eligible DTI data. Besides, participants with complete baseline data and at least one follow-up cognitive assessments data and eligible DTI data were used for longitudinal analyses. A detailed flowchart illustrating participants inclusion and exclusion can be found in Fig. 1. In addition, we also performed a subgroup analysis based on the Aβ status. Aβ positivity (Aβ + ) was defined in CSF (CSF Aβ42 < 976 pg/ml) or on amyloid PET (18F-Florbetapir ≥ 1.11 or 18F-Florbetaben ≥ 1.08) as described previously [22, 23]. A total of 764 Aβ+ participants were finally included in subgroup analysis.

### Cognitive function assessment

To evaluate global cognitive function, we employed baseline and follow-up results from the Mini-Mental State Examination (MMSE) [24]. The MMSE serves as a concise cognitive screening tool, utilizing a scoring scale ranging from 0 to 30. Widely applied for the detection and ongoing assessment of dementia progression, it provides valuable insights into the severity of cognitive impairment. The slopes of change in MMSE scores (ΔMMSE scores) were determined for participants with longitudinal MMSE data through the implementation of Linear Mixed Effects (LME) models. These models incorporated both random intercepts and slopes, accounting for individual variability in baseline scores and the trajectories of change over time. This analytical approach was applied to explore the longitudinal cognitive changes in the study cohort.

### MRI data acquisition

The T1-weighted (T1W) imaging data were acquired for each participant with 3D T1W Inversion Recovery prepared Spoiled Gradient Recalled echo sequence or 3D T1W magnetization prepared rapid gradient echo sequence on a 3 T scanner. Diffusion tensor imaging (DTI) was performed with echo planar imaging sequence. Detailed imaging acquisition is available on the ADNI website (https://adni.loni.usc.edu/methods/documents/mri-protocols/).

### CP segmentation

All T1W images were first processed with Freesurfer 7.3 to obtain CP volume and total intracranial volume (ICV) using the recon-all pipeline. In order to obtain more accurate results of CP volume, we next applied a lightweight segmentation algorithm based on the Gaussian Mixture Models (GMM), which showed better performance than Freesurfer [25]. CPV was expressed as the ratio of CP volume to the total ICV (ratio of ICV × 10^3^). To assess inter-method reliability, manual segmentation of CP was performed in a subset of 30 participants. An experienced neuroradiologist blinded to clinical data manually delineated using ITKsnap (http://www.itksnap.org). As expected, strong Pearson correlation confirmed a good consistency between GMM and manual segmentation (r = 0.70, *p* < 0.001).

### White matter microstructural assessment

The peak width of skeletonized mean diffusivity (PSMD) served as an indicator to assess global white matter microstructural damage [26]. Previous research indicates the PSMD can detect more nuanced changes in white matter integrity beyond conventional markers and is also strongly correlated with cognitive function [26–29]. PSMD was calculated using a fully automated pipeline (www.psmd-marker.com/) [26]. Initially, DTI images underwent preprocessing using FSL version 6.0, including eddy current and motion correction. The PSMD computation utilized the PSMD marker script alongside FSL. The workflow incorporated essential steps: first, white matter tract skeletonization was achieved through tract-based spatial statistics (TBSS) using a 1 mm fractional anisotropy (FA) template at a threshold FA value of 0.2. Subsequently, mean diffusivity (MD) images were projected onto the skeleton, guided by FA-derived parameters. To address potential contamination from CSF, the MD skeletons were carefully masked with a standard skeleton thresholded at 0.3. PSMD was then calculated as the difference between the 5th and 95th percentiles of voxel-based MD values within the refined white matter skeleton.

### Glymphatic function assessment

The evaluation of glymphatic function in vivo encountered challenges primarily stemming from the requisite intrathecal or intravenous administration of tracers and the need for multiple imaging sessions to monitor fluid dynamics. These constraints persisted until a novel method, DTI-analysis along the perivascular space (DTI-ALPS), was employed for the non-invasively assess glymphatic function [30]. A recent study found a strong correlation between the DTI-ALPS and glymphatic clearance in the brain, as measured by standard contrast-enhanced MRI [31]. This indicates that DTI-ALPS can serve as a practical proxy for brain glymphatic clearance. The DTI-ALPS was calculated follow a fully automated FSL pipeline previously described [32]. The workflow mainly includes the following steps: (1) corrected images for artifacts using Marchenko-Pastur Principal Component Analysis (MP-PCA) denoising and Gibbs unringing by MRtrix3; (2) Addressed eddy current, motion correction and skull-stripped with FSL; (3) Generated FA map and three diffusivity maps along the x-axis (D_xx_), y-axis (D_yy_), and z-axis (D_zz_) using the dtifit function; (4) Co-registered FA maps to the FA map template by using linear registration, and applied the registration matrix to other diffusivity maps; (5) Identified the superior corona radiata (SCR) and the superior longitudinal fasciculus (SLF) based on the JHU DTI-based white matter atlas, and defined 5 mm diameter sphere masks in bilateral SCR and SLF areas for subsequent extraction of the mean diffusivity values (D_xx_, D_yy_, D_zz_) utilized in ALPS calculation.

### Systemic inflammation assessment

Elevated NLR is commonly observed in AD patients and can reflect the severity of systematic inflammation [33]. Peripheral blood was collected after an overnight fasting, and analyzed using automated equipment and standard methods.

### Statistical analysis

Data were reported using median (IQR) or presented as counts and percentages (%) in accordance with standard statistical conventions. One-way analysis of variance (one-way ANOVA) was employed to assess the disparities in continuous variables across distinct groups, while the chi-square test was utilized for categorical variables. Pairwise comparisons between groups were conducted using the Tukey correction to account for multiple comparisons and ensure robust statistical inference.

We used generalized linear models (GLM) to analyze differences in CPV, PSMD, DTI-ALPS, and NLR between groups controlled for age, sex, education and APOE4 carrier status. Post-hoc analyses were conducted with the incorporation of Tukey correction for multiple comparisons.

Multiple linear regression was employed to assess the relationship among CPV, PSMD and cognitive function, as well as the longitudinal effects of CPV and PSMD on cognitive progress. Additionally, mediation analyses were performed using the mediation package in R to further explore the aforementioned associations. The bootstrap method with 5000 samples was employed to estimate coefficients of indirect effects while controlling for the same covariates as mentioned above.

Finally, we performed a partial correlation analysis to assess the association of CPV with NLR and DTI-ALPS while controlling the aforementioned covariates.

The above analyses were performed in both the whole cohort and Aβ+ participants. All statistical analyses were performed using R version 4.2.2 and SPSS 25.0, and statistical significance was set at *p* < 0.05 (two tailed).

## Results

### Clinical and demographic characteristics

The baseline demographic characteristics of participants were summarized in Table 1. Among 1351 cognitively impaired participants: 344 with SCD (38.1% male; mean age, 70.69 ± 6.75 years), 761 with MCI (55.2% male; mean age, 71.82 ± 7.56 years), 246 with AD (56.5% male; mean age, 74.36 ± 8.19 years). Significant differences were observed between the three groups in gender (*p* < 0.001), age (*p* < 0.001), education (*p* < 0.001), Aβ+ participants count (*p* < 0.001), and the APOE4 carrier status (*p* < 0.001). Additionally, a progressive elevation in CPV (*p* < 0.001), NLR (*p* < 0.001), and PSMD (*p* < 0.001), coupled with a gradual reduction in DTI-ALPS (*p* < 0.001) was observed with cognitive deterioration.

**Table 1:** Clinical and demographic characteristics of participants according to disease stage.

	SCD	MCI	AD	*P* Value			
	*N* = 344	*N* = 761	*N* = 246	SCD vs MCI vs AD	SCD vs MCI	SCD vs AD	MCI vs AD
Sex (male)	131 (38.1%)	420 (55.2%)	139 (56.5%)	<0.001	<0.001	<0.001	0.719
Age	70.69 ± 6.75	71.82 ± 7.56	74.36 ± 8.19	<0.001	0.050	<0.001	<0.001
Education	16.67 ± 2.33	16.12 ± 2.64	15.51 ± 2.73	<0.001	0.003	<0.001	0.003
Aβ positive	134 (41.6%) (322)	427(60.5%) (706)	203 (89.4%) (227)	<0.001	<0.001	<0.001	<0.001
APOE4 carrier	102 (36.2%) (282)	317(47.2%) (671)	153 (68.0%) (225)	<0.001	0.005	<0.001	<0.001
CPV	1.42 ± 0.42	1.57 ± 0.46	1.88 ± 0.57	<0.001	<0.001	<0.001	<0.001
NLR	2.22 ± 0.91 (253)	2.41 ± 1.18 (746)	2.71 ± 1.23 (223)	<0.001	0.049	<0.001	0.002
DTI-ALPS	1.21 ± 0.14 (263)	1.18 ± 0.16 (322)	1.10 ± 0.16 (115)	<0.001	0.029	<0.001	<0.001
PSMD 10^−4^	2.56 ± 0.82 (263)	2.89 ± 0.85 (322)	3.38 ± 1.11 (115)	<0.001	<0.001	<0.001	<0.001
MMSE	29.07 ± 1.17	27.83 ± 1.86	23.02 ± 2.30	<0.001	<0.001	<0.001	<0.001

*SCD* subjective cognitive decline, *MCI* mild cognitive impairment, *AD* Alzheimer’s disease, *CP* choroid plexus volume, *NLR* neutrophil-lymphocyte ratio, *DTI-ALPS* DTI-analysis along the perivascular space, *PSMD* peak width of skeletonized mean diffusivity, *MMSE* mini-mental state examination.

### Between-group differences in CPV, PSMD, DTI-ALPS and NLR

**Fig. 2:**
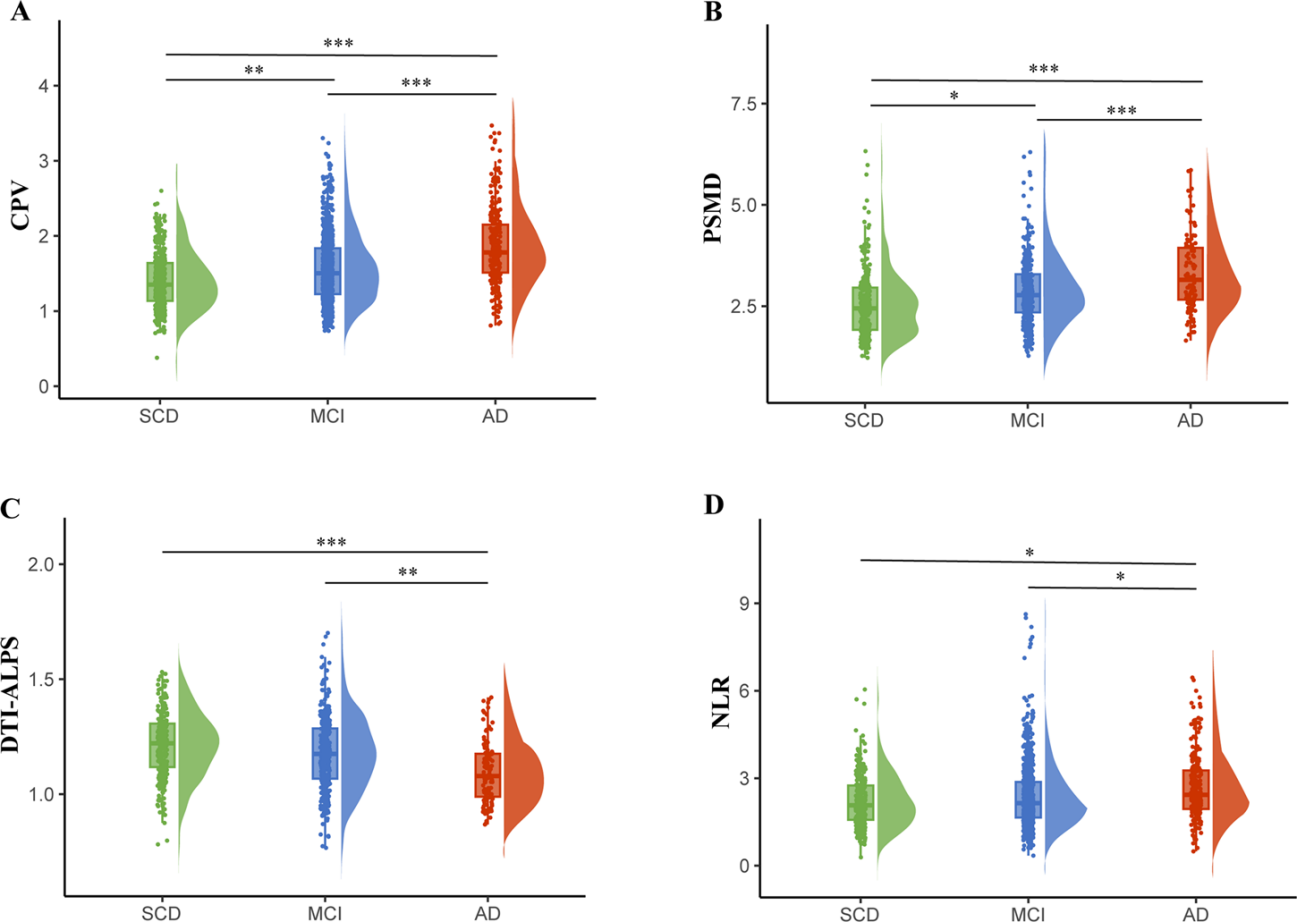
Rain-cloud plots show CPV, PSMD, DTI-ALPS and NLR comparison between groups. CPV (**A**), PSMD (**B**), and NLR (**D**) were significantly higher and DTI-ALPS (**C**) was significantly lower in participants with AD than SCD. *, corrected *p* < 0.05; **, corrected *p* < 0.01; ***, corrected *p* < 0.001. SCD subjective cognitive decline, MCI mild cognitive impairment, AD Alzheimer’s disease, CPV choroid plexus volume, NLR neutrophil-lymphocyte ratio, DTI-ALPS DTI-analysis along the perivascular space, PSMD peak width of skeletonized mean diffusivity.

In the overall sample, following adjustments for age, sex, education, and APOE4 carrier status, DTI-ALPS (*p* < 0.001) was significantly lower and CPV (*p* < 0.001), PSMD (*p* < 0.001), and NLR (*p* = 0.035) were significantly higher in participants with AD than SCD. Furthermore, CPV (*p* = 0.001) and PSMD (*p* = 0.016) were significantly higher in the participants with MCI than SCD. Similar to the comparison between AD and SCD, DTI-ALPS (*p* = 0.003) was significantly lower and CPV (*p* < 0.001), NLR (*p* = 0.023), and PSMD (*p* < 0.001) were significantly higher in participants with AD than MCI (Fig. 2).

In the Aβ+ participants, significantly higher CPV (*p* < 0.001), NLR (*p* = 0.048), and PSMD (*p* < 0.001) was still observed in the participants with AD than SCD. DTI-ALPS (*p* = 0.009) was remained significantly lower and CPV (*p* < 0.001), PSMD (*p* < 0.001) were higher in participants with AD than MCI. Conversely, significant differences in CPV and PSMD observed between participants with MCI and those with SCD in the total sample were no longer evident (Supplementary Figure 1).

### Relationship among CPV, PSMD, and cognitive function

**Fig. 3:**
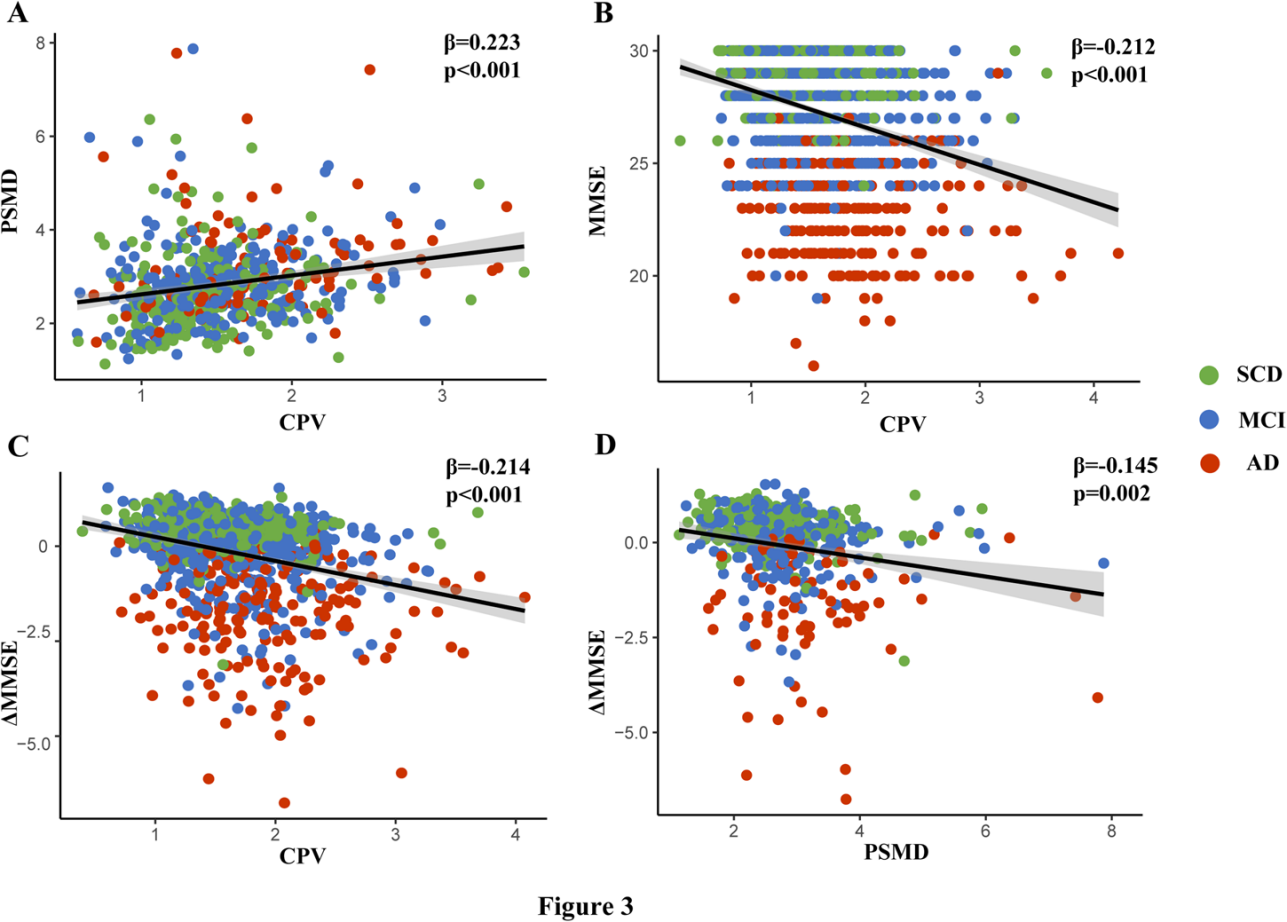
Scatter plots show the association of CPV with PSMD and MMSE in both cross-sectional and longitudinal studies. CP enlargement was independently correlated with PSMD (**A**) and MMSE (**B**) in the cross-sectional study. CP enlargement (**C**) and PSMD (**D**) were independently correlated with longitudinal changes in MMSE. β, multivariable linear regression standardized β. SCD subjective cognitive decline, MCI mild cognitive impairment, AD Alzheimer’s disease, CPV choroid plexus volume, PSMD peak width of skeletonized mean diffusivity, MMSE Mini-Mental State Examination.

**Fig. 4:**
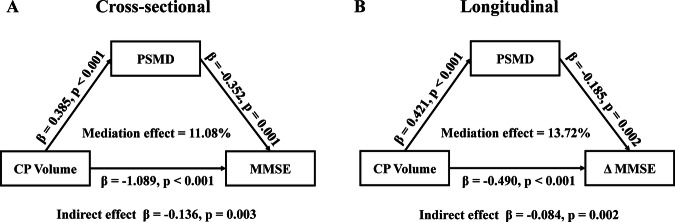
Partial mediation effect of PSMD between CPV and MMSE in both cross-sectionally and longitudinally. **A** The PSMD partially mediated the association between CPV and cognitive function. **B** The PSMD partially mediated the association between CPV and cognitive progress. CP choroid plexus, PSMD peak width of skeletonized mean diffusivity, MMSE Mini-Mental State Examination.

In the overall sample, multivariate linear regression model revealed that CPV was significantly associated with PSMD (β = 0.223, *p* < 0.001; Table 2; Fig. 3A) after adjusting for age, sex, education, and APOE4 carrier status. Meanwhile, a statistically significant association between CPV and MMSE was found (β = −0.212, *p* < 0.001; Table 2; Fig. 3B) after adjusting for PSMD and the above covariates. We further conducted a mediation analysis to investigate the complex relationship between CPV, PSMD, and MMSE. The results showed the PSMD partially mediated the association between CPV and MMSE after adjusting for covariates (indirect effect β = −0.136, *p* = 0.003, mediation effect = 11.08%; Fig. 4A).

**Table 2:** Multivariate linear regression analysis of CPV on PSMD and cognitive function.

Variables	B	95% CI		β	*P* value
		Lower	Upper		
**Model 1: PSMD**					
Age	0.020	0.009	0.032	0.158	<0.001
Sex	0.138	−0.016	0.292	0.074	0.079
Education	0.008	−0.022	0.039	0.022	0.585
APOE4 carrier	0.051	−0.064	0.167	0.036	0.385
CPV	0.404	0.250	0.558	0.223	<0.001
**Model 2: MMSE**					
Age	−0.040	−0.071	−0.009	−0.106	<0.011
Sex	−0.333	−0.760	−0.094	−0.060	0.126
Education	0.292	0.208	0.376	0.263	<0.001
APOE4 carrier	−0.843	−1.162	−0.523	−0.203	<0.001
CPV	−1.128	−1.564	−0.693	−0.212	<0.001
PSMD	−0.398	−0.632	−0.164	−0.136	0.001

*CPV* choroid plexus volume, *PSMD* peak width of skeletonized mean diffusivity, *MMSE* Mini-Mental State Examination, *CI* confidence interval, *B* unstandardized regression coefficient, *β* standardized regression coefficient.

In the Aβ+ participants, similar to the above results, the larger CPV was associated with higher PSMD (β = 0.225, *p* < 0.001; Supplementary Table 1; Supplementary Figure 2A) and lower MMSE (β = −0.229, *p* < 0.001; Supplementary Table 1; Supplementary Figure 2B) after adjusting for the above covariates. In addition, the mediation analysis revealed that PSMD played a significant role in mediating the relationship between CPV and MMSE (indirect effect β = −0.158, *p* = 0.021, mediation effect = 12.16%; Supplementary Figure 3A).

### Longitudinal effects of CPV and PSMD on cognitive progress

Among 511 participants with longitudinal data, baseline CP enlargement (β = −0.214, *p* < 0.001; Table 3; Fig. 3C) and higher PSMD (β = −0.145, *p* = 0.002; Table 3; Fig. 3D) were independently associated with more rapid cognitive decline quantified with ΔMMSE (β = −0.217, *p* < 0.001; Table 3; Fig. 3D). Mediation analysis subsequently showed that PSMD significantly mediated the associations between baseline CPV and ΔMMSE (indirect effect β = −0.084, *p* = 0.002, mediation effect = 13.72%; Fig. 4B).

**Table 3:** Longitudinal effects of CPV and PSMD on cognitive progress.

Variables	B	95% CI		β	*P* value
		Lower	Upper		
**Model: ΔMMSE**					
Age	−0.010	−0.026	0.006	−0.060	0.205
Sex	−0.011	−0.220	0.198	−0.004	0.920
Education	0.076	0.036	0.117	0.161	<0.001
APOE4 carrier	−0.415	−0.573	−0.257	−0.228	<0.001
CPV	−0.485	−0.696	−0.273	−0.214	<0.001
PSMD	−0.187	−0.303	−0.071	−0.145	0.002

*CPV* choroid plexus volume, *PSMD* peak width of skeletonized mean diffusivity *MMSE* mini-mental state examination, *CI* confidence interval, *B* unstandardized regression coefficient, *β* standardized regression coefficient.

In the Aβ+ participants, CPV (β = −0.235, *p* < 0.001; Supplementary Table 2; Supplementary Figure 2C) and PSMD (β = −0.148, *p* = 0.017; Supplementary Table 2; Supplementary Figure 2D) were still significantly associated with ΔMMSE. Moreover, the PSMD partially mediated the association between baseline CPV and cognitive progress (indirect effect β = −0.088, *p* = 0.025, mediation effect = 13.15%; Supplementary Figure 3B).

### Association of CPV with NLR and DTI-ALPS

**Fig. 5:**
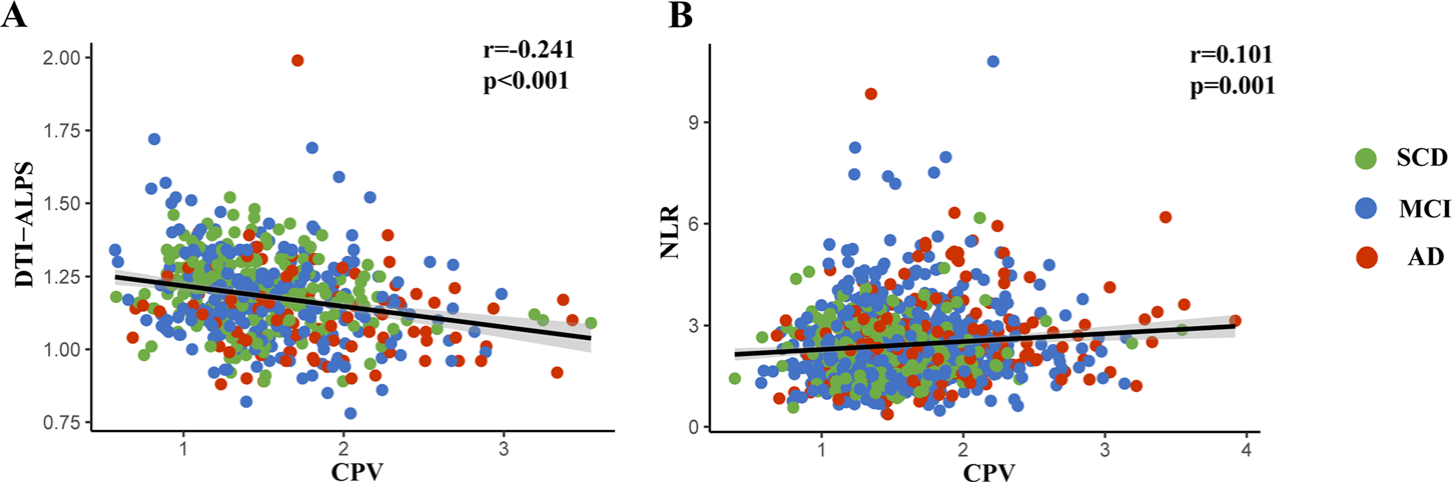
Scatter plots show the association of CPV with DTI-ALPS and NLR. CP enlargement was significantly associated with **A** lower DTI-ALPS and **B** higher NLR. SCD subjective cognitive decline, MCI mild cognitive impairment, AD Alzheimer’s disease, CPV choroid plexus volume, NLR neutrophil-lymphocyte ratio, DTI-ALPS DTI-analysis along the perivascular space.

Using partial correlation analysis adjusted for age, sex, education, and APOE4 carrier status, the larger CPV was significantly associated with higher NLR (r = 0.101, *p* = 0.001) and lower DTI-ALPS (r = −0.241, *p* < 0.001) in the overall sample (Fig. 5). Similar results were also found for the association of CPV with NLR (r = 0.085, *p* = 0.031) and DTI-ALPS (r = −0.271, *p* < 0.001) in the Aβ+ participants (Supplementary Figure 4).

## Discussion

The study investigated the relationship between CPV, white matter microstructural change, cognitive function, glymphatic function, and systemic inflammation in AD. The main findings are summarized as follows: (1) with the progression of cognitive impairment, a gradual enlargement in CPV was observed; (2) CP enlargement was independently correlated with PSMD and MMSE in both cross-sectional and longitudinal studies. Furthermore, our findings suggested that impaired white matter microstructure partially mediated the effect of CP enlargement on both severity and progression of cognitive function; (3) CP enlargement was associated with impaired glymphatic function and higher systemic inflammation.

While most attention has historically focused on gray matter due to its housing of neuronal cell bodies, the significance of white matter in AD has been somewhat overlooked. Recent neuroimaging research has unveiled a correlation between microstructural and macrostructural abnormalities in white matter and the risk and progression of AD, emphasizing its underestimated role [34–36]. Additionally, axonal damage reflected by circulating neurofilament light chain (NfL) has been correlated with cognitive impairment [1]. This suggests that, beyond the neuronal pathological features of the disease, white matter degeneration and demyelination also constitute significant pathophysiological characteristics [37]. Notably, a recent animal study showed disturbances in myelin and demyelination-associated genetic pathways serve as potent drivers of amyloid deposition in AD mouse models, and these processes may contribute to plaque formation by weakening the protective function of microglia against plaques [38]. Our recent study showed that GLP-1R agonist mitigates Alzheimer-related phenotypes, while GLP-1R agonists may have beneficial effect on myelin formation and preventing demyelination [39, 40]. This underscores the potential therapeutic importance of preserving myelin integrity and enhancing oligodendrocyte function in AD. The PSMD utilized in our study is an automated quantitative imaging marker for assessing global white matter microstructural damage and can predict cognitive outcomes in neurological disorders [41]. Our results revealed a gradual increase in PSMD with the progression of cognitive impairment, signifying a progressive deterioration in white matter microstructure. Moreover, further analysis revealed a pronounced mediating effect of white matter microstructure on the association between CPV and cognitive function in both cross-sectional and longitudinal evaluations. These findings highlight the importance of CP and white matter in AD. Nevertheless, we can currently only speculate about the pathophysiological mechanisms through which CP enlargement affects changes in white matter microstructure. CP enlargement may be attributed to factors such as atrophy of CP epithelial cells, interstitial fibrosis, stromal dystrophic calcification, thickening of blood vessels walls and basement membrane [15, 42]. These changes may disrupt the production and clearance of CSF [43]. Disruptions in CSF dynamics may result in the deposition of neurotoxic waste products and inflammatory factors, subsequently causing damage in white matter [44]. Simultaneously, the involvement of CP in brain lipid metabolism and circadian rhythm regulation may also contribute to white matter impairment [45–47]. While our study elaborately delineates the relationship between CPV, PSMD, and cognition, the specific mechanisms still require further clarification.

The glymphatic system serves as the waste drainage system in the brain, involving the movement of CSF through perivascular and interstitial spaces, and subsequently exits along veins [48]. Emerging evidence underscores the critical role of the glymphatic system in clearing Aβ and tau proteins in AD [49, 50]. Additionally, risk factors associated with AD, including aging, neurovascular damage, and sleep disorders, are all linked to the glymphatic system dysfunction [51–53]. Our study also revealed a significant decrease in DTI-ALPS in AD patients. Previous studies have suggested that AQP4 and arterial pulsation exert influence on the glymphatic circulation [54, 55]. CSF and interstitial fluid together constitute essential pathways for the glymphatic circulation. Since CSF is primarily produced by CP, we hypothesize a potential correlation between CPV and glymphatic system. It is noteworthy that a significant correlation was observed between CP enlargement and reduced DTI-ALPS in our study. This suggested that alteration in CSF production may have a discernible impact on glymphatic activity. However, the causal relationship between them remains unclear. It is plausible that CP enlargement may lead to reduced CSF secretion, resulting in diminished glymphatic circulation. Conversely, a decrease in glymphatic circulation may trigger compensatory enlargement of the CP. Regardless of the underlying mechanisms, it is evident that CPV play a crucial role in glymphatic system and CP enlargement reflects impairment in cerebral waste clearance function in AD. Elucidating the relationship between these changes is essential for understanding the pathophysiological mechanisms of AD. Further animal experiments are required to establish causation definitively.

Inflammation plays a crucial role in the onset and progression of AD, and inflammation-related genes have been implicated in the pathological changes and cognitive dysfunction observed in AD [56, 57]. CP enlargement is not only observed in AD but also in other neuroinflammatory diseases such as multiple sclerosis and Parkinson disease [58, 59]. Therefore, some studies proposed CPV could be served as a potential marker of cerebral inflammation [60]. Given the fenestrated nature of capillaries in the CP, which results in the absence of a blood-brain barrier (BBB), immune cells situated within the stroma of the CP are further exposed to external stimuli from the periphery [61]. Previous mouse study suggested that CP acts as a trigger for initiating inflammatory responses within the brain under peripheral immune stimulation [62]. A recent study on patients with MS indicated that CP enlargement may occur prior to pathological changes in the brain [63]. Our analysis also revealed an association between peripheral systemic inflammation and CPV. These findings above suggested that peripheral inflammation may contribute to CP enlargement. One possible mechanism is prolonged peripheral systemic inflammation results in the infiltration of immune cells into the CP, leading to its morphological and functional changes [64]. Simultaneously, inflammatory cells enter the CSF and brain parenchyma, disrupting the brain homeostatic environment and inducing a pro-inflammatory state [65, 66]. Ultimately, this cascade of events contributes to a decline in cognitive function.

Our findings suggested that CPV could serve as a promising non-invasive approach to reflect a spectrum of pathophysiological changes associated with AD, including white matter microstructure, glymphatic function, peripheral systemic inflammation, and cognitive function. Simultaneously, these results support the consideration of intervening in peripheral systemic inflammation and CP as potential therapeutic targets for AD. In AD mouse models, previous studies have demonstrated the aggregation of neutrophils at sites of brain capillary adhesion, leading to the disruption of the BBB [67]. Serum VEGF-A was found to reduce cognitive damage in AD mice by hindering the invasion of neutrophils into the brain [68]. Additionally, the use of anti-Ly6G antibodies to diminish neutrophil accumulation and adhesion significantly enhances cerebral blood flow, thereby improving spatial short-term memory [69]. The CP primarily comprises CP epithelial cells (CPECs) situated in the lateral ventricles of the brain, can serve as a more promising therapeutic target for AD. Previous research has indicated that overexpression of CYP46A1 in the CP can enhance cognitive function and reduce brain inflammation in mice [70]. Given that CPECs contain a subpopulation of neural progenitor cells capable of proliferation and differentiation into various cell types, the transplantation of CPECs into the brains of AD mouse models has shown a significant reduction in brain Aβ deposition, tau phosphorylation, and reactive astrocytosis [71]. The above findings suggested that targeting peripheral systemic inflammation and the CP is a promising direction. Due to the significant advantage of the CP in traversing the BBB, further exploration into peripheral systemic inflammation and the CP holds the potential for unexpected breakthroughs in AD therapy.

Several limitations should be acknowledged in our study. First, our study employed the GMM automatic segmentation method for CP, which may not fully capture its true structure and size. Given the relatively large sample size, we still chose to proceed with this method. Although subgroup analysis revealed a good inter-method reliability compared to manual segmentation, it’s important to note that manual segmentation remains the gold standard for obtaining CPV. Secondly, the DTI-ALPS utilized in this study reflects perivascular clearance activity and may not comprehensively depict the entire process of the glymphatic system. Future studies employing traditional contrast-based dynamic methods are recommended for a more in-depth understanding of glymphatic function. Finally, the cross-sectional correlation analysis between CPV and the glymphatic circulation, as well as peripheral system inflammation, could not determine a causal relationship. Subsequent longitudinal imaging studies were needed to ascertain the dynamic changes in their relationship over time.

## Conclusion

Our study demonstrated that CPV gradually increased with the progression of cognitive impairment. The CP enlargement reflected various pathophysiological changes during the course of AD (white matter microstructure, glymphatic function, peripheral systemic inflammation, and cognitive function). Additionally, it served as an early indicator of white matter microstructural damage and accelerated cognitive decline. Thus, CPV holds promise as a diagnostic, monitoring, and therapeutic target in AD. Further longitudinal human trials and animal studies are warranted to validate our findings and elucidate the underlying molecular mechanisms.

## Supplementary information

Supplementary material

Consortium author list

## Data Availability

The datasets generated and/or analyzed during the current study are available in the ADNI study. More details in http://www.adni-info.org.
