# Supplementary material for: Association of choroid plexus volume with white matter microstructure, glymphatic function, and peripheral systemic inflammation in Alzheimer’s disease
Source: Transl Psychiatry. 2025 Jul 11;15:238. doi: 10.1038/s41398-025-03432-1 (PMC12254307; doi:10.1038/s41398-025-03432-1)
Supplement: Supplementary file 1 — Supplementary material [file 41398_2025_3432_MOESM1_ESM.docx]

**Association of choroid plexus volume with white matter microstructure, glymphatic function, and peripheral systemic inflammation in Alzheimer's disease**

**Supplementary material**

Supplemental Figures and Tables

Figures S1-4, Tables S1-2


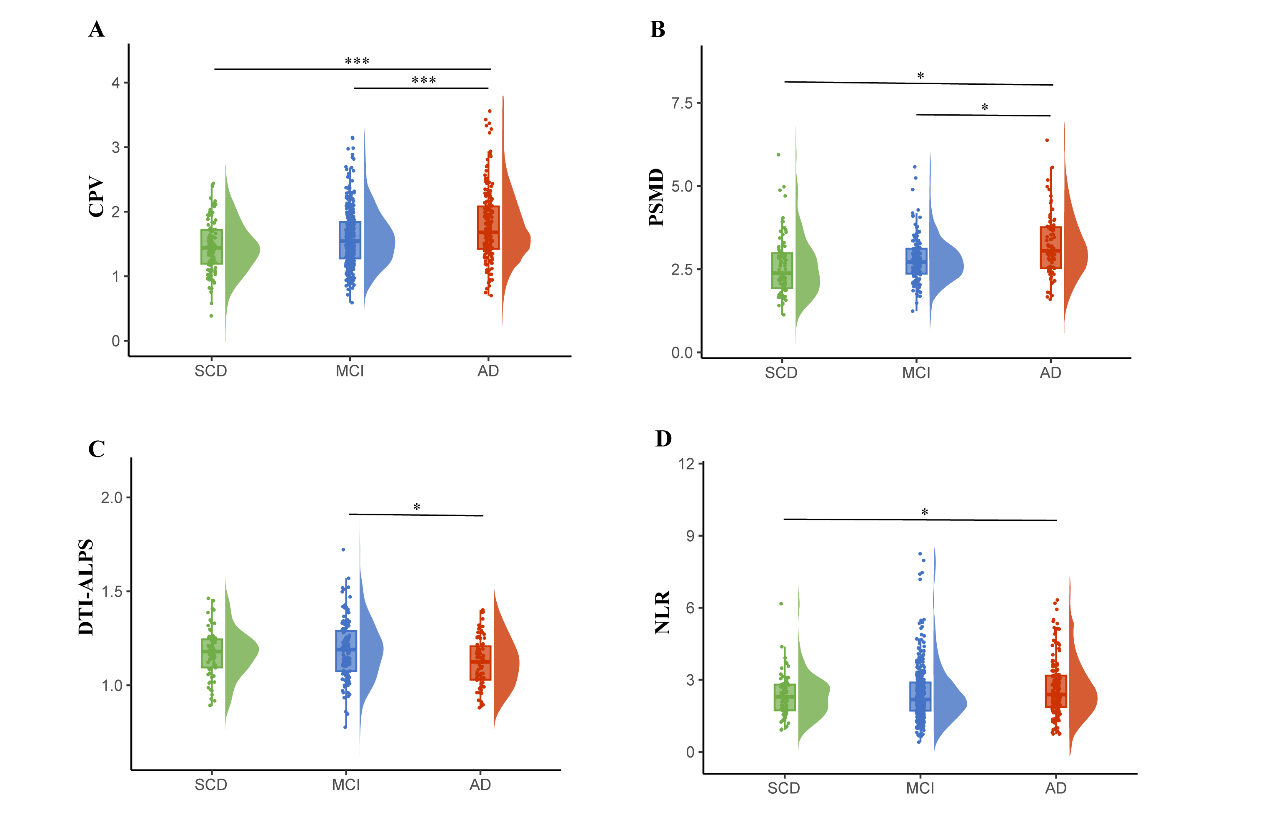


**Supplementary Figure 1. Rain-cloud plots show CPV, PSMD, DTI-ALPS and NLR comparison between groups in Aβ+ participants.** Significantly higher CPV, NLR, and PSMD observed in the participants with AD than SCD. *, corrected p<0.05; **, corrected p<0.01; ***, corrected p<0.001. SCD, subjective cognitive decline; MCI, mild cognitive impairment; AD, Alzheimer’s disease; CPV, choroid plexus volume; NLR, neutrophil-lymphocyte ratio; DTI-ALPS, DTI-analysis along the perivascular space; PSMD, peak width of skeletonized mean diffusivity.


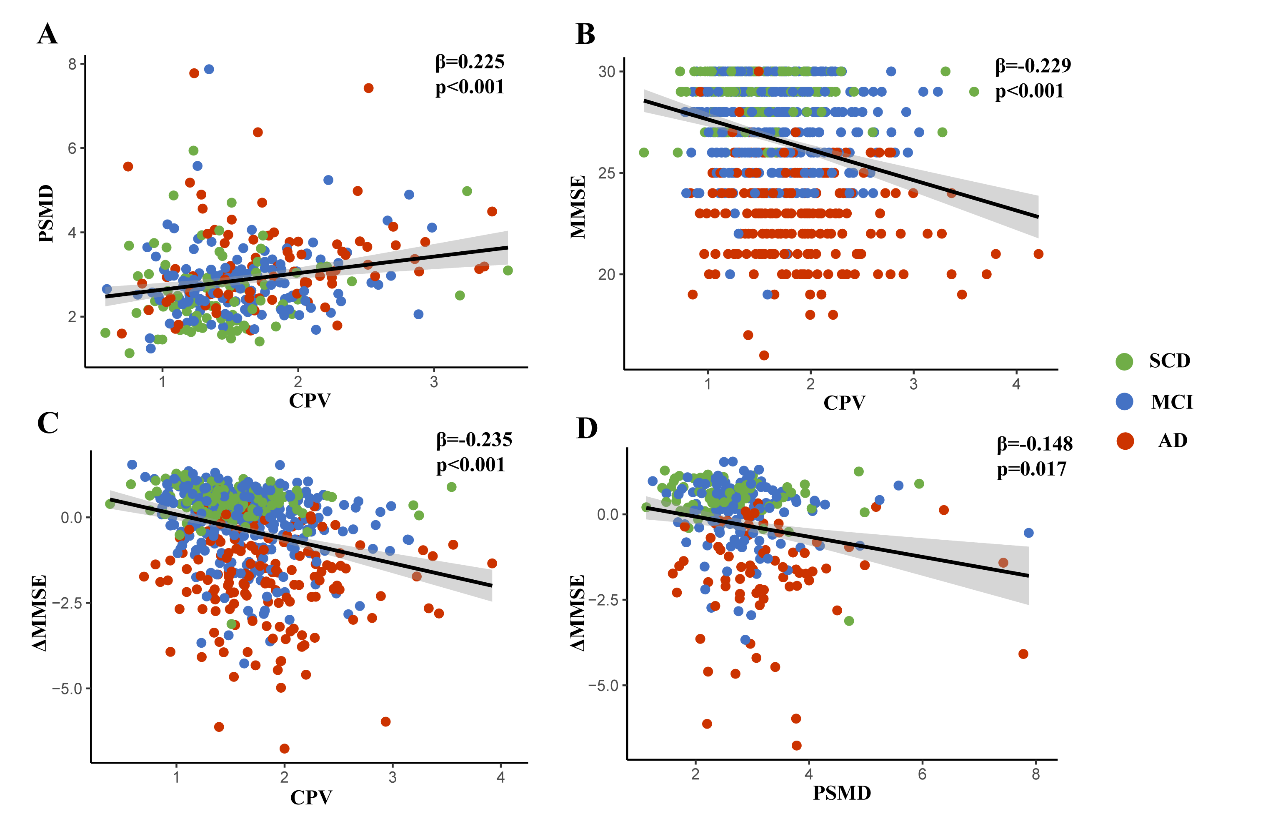


**Supplementary Figure 2. Scatter plots show the association of CPV with PSMD and MMSE in both cross-sectional and longitudinal studies in Aβ+ participants.** CP enlargement was independently correlated with PSMD and MMSE in both cross-sectional and longitudinal studies. β, multivariable linear regression standardized β. SCD, subjective cognitive decline; MCI, mild cognitive impairment; AD, Alzheimer’s disease; CPV, choroid plexus volume; PSMD, peak width of skeletonized mean diffusivity; MMSE, Mini-Mental State Examination.


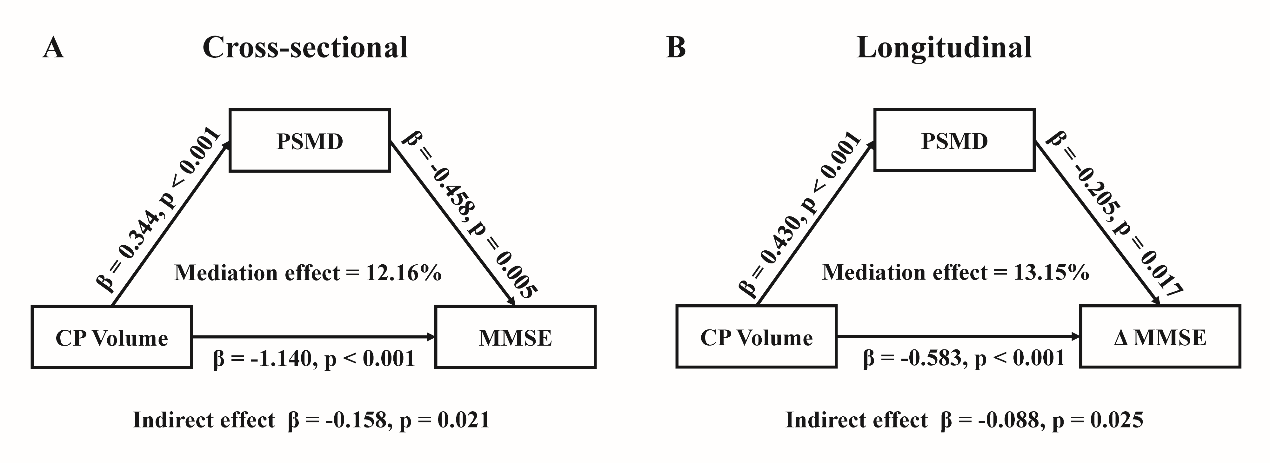


**Supplementary Figure 3. Partial mediation effect of PSMD between CPV and MMSE in both cross-sectionally and longitudinally in Aβ+ participants.** (A) The PSMD partially mediated the association between CPV and cognitive function. (B) Change in PSMD partially mediated the association between CPV and cognitive progress. CP, choroid plexus; PSMD, peak width of skeletonized mean diffusivity; MMSE, Mini-Mental State Examination.


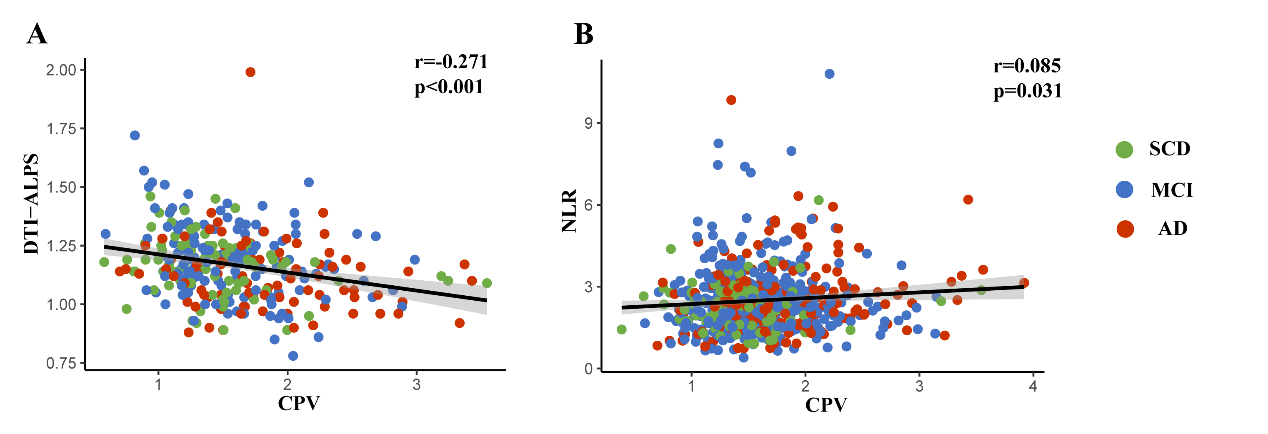


**Supplementary Figure 4. Scatter plots show the association of CPV with DTI-ALPS and NLR in Aβ+ participants.** CP enlargement was significantly associated with (A) lower DTI-ALPS and (B) higher NLR. SCD, subjective cognitive decline; MCI, mild cognitive impairment; AD, Alzheimer’s disease; CPV, choroid plexus volume; NLR, neutrophil-lymphocyte ratio; DTI-ALPS, DTI-analysis along the perivascular space.

**Supplementary Table 1. Multivariate linear regression analysis of CPV on PMSD and cognitive function in Aβ+ participants**

| **Variables** | **B** | **95% CI** | | **β** | **P value** |
| --- | --- | --- | --- | --- | --- |
|  |  | **Lower** | **Upper** |  |  |
| **Model 1: PSMD** |  |  |  |  |  |
| Age | 0.015 | -0.001 | 0.031 | 0.113 | 0.068 |
| Sex | 0.157 | -0.062 | 0.376 | 0.080 | 0.159 |
| Education | 0.002 | -0.039 | 0.044 | 0.006 | 0.908 |
| APOE4 carrier | 0.053 | -0.110 | 0.216 | 0.037 | 0.525 |
| CPV | 0.395 | 0.194 | 0.595 | 0.225 | <0.001 |
| **Model 2: MMSE** |  |  |  |  |  |
| Age | -0.018 | -0.067 | 0.030 | -0.044 | 0.459 |
| Sex | -0.182 | -0.841 | 0.477 | -0.030 | 0.587 |
| Education | 0.273 | 0.149 | 0.379 | 0.231 | <0.001 |
| APOE4 carrier | -0.515 | -1.005 | -0.026 | -0.116 | <0.039 |
| CPV | -1.251 | -1.867 | -0.635 | -0.229 | <0.001 |
| PSMD | -0.499 | -0.838 | -0.160 | -0.160 | 0.004 |

**Abbreviations:** Aβ+, β-amyloid positivity; CPV, choroid plexus volume; PSMD, peak width of skeletonized mean diffusivity; MMSE, Mini-Mental State Examination; CI, confidence interval.

**Supplementary Table 2. Longitudinal effects of CPV and PMSD on cognitive progress in Aβ+ participants**

| **Variables** | **B** | **95% CI** | | **β** | **P value** |
| --- | --- | --- | --- | --- | --- |
|  |  | **Lower** | **Upper** |  |  |
| **Model: ΔMMSE** |  |  |  |  |  |
| Age | 0.007 | -0.018 | 0.032 | 0.037 | 0.574 |
| Sex | 0.068 | -0.263 | 0.399 | 0.024 | 0.686 |
| Education | 0.068 | 0.005 | 0.130 | 0.126 | 0.034 |
| APOE4 carrier | -0.244 | -0.493 | 0.004 | -0.119 | 0.054 |
| CPV | -0.576 | -0.888 | -0.265 | -0.235 | <0.001 |
| PSMD | -0.208 | -0.379 | -0.037 | -0.148 | 0.017 |

**Abbreviations:** Aβ+, β-amyloid positivity; CPV, choroid plexus volume; PSMD, peak width of skeletonized mean diffusivity; MMSE, Mini-Mental State Examination; CI, confidence interval.
