# Supplementary material for: Association of choroid plexus volume with white matter microstructure, glymphatic function, and peripheral systemic inflammation in Alzheimer’s disease
Source: Transl Psychiatry. 2025 Jul 11;15:238. doi: 10.1038/s41398-025-03432-1 (PMC12254307; doi:10.1038/s41398-025-03432-1)
Supplement: Supplementary file 2 — Consortium author list [file 41398_2025_3432_MOESM2_ESM.pdf]

ADNI Alzheimer's Disease Neuroimaging Initiative

ACKNOWLEDGEMENT LIST FOR ADNI PUBLICATIONS

The Data and Publications Committee, in keeping with the publication policies adopted by the ADNI Steering Committee, here provide lists for standardized acknowledgement. The list consists of two parts: Infrastructure Investigators and Site Investigators. Infrastructure Investigators represent the names responsible for leadership and infrastructure. Site Investigators represent the names of individuals at each recruiting site. All papers, including methodological papers, should have an acknowledgement list that consists of Infrastructure Investigators plus the FULL list.

I. ADNI 1, GO, 2, 3, 4

Part A: Leadership and Infrastructure

Principal Investigator

Michael Weiner, MD	University of California, San Francisco<br>Northern California Institute for Research and Education

ATRI PI and Director of Coordinating Center Clinical Core

Paul Aisen, MD	University of Southern California
Ronald Petersen, MD, PhD	Mayo Clinic, Rochester (co-PI of of Clinical Core)

Executive Committee

Michael Weiner, MD	University of California, San Francisco
Paul Aisen, MD	University of Southern California
Ronald Petersen, MD, PhD	Mayo Clinic, Rochester
Clifford R. Jack, Jr., MD	Mayo Clinic, Rochester
William Jagust, MD	University of California, Berkeley
Susan Landau, PhD	University of California, Berkeley
Monica Rivera-Mindt, PhD	Fordham University; Mt. Sinai Medical Center
Ozioma Okonkwo, PhD	University of Wisconsin
Leslie M. Shaw, PhD	University of Pennsylvania
Edward B. Lee, MD, PhD	University of Pennsylvania
Arthur W. Toga, PhD	University of California, Los Angeles
Laurel Beckett, PhD	University of California, Davis
Danielle Harvey, PhD	University of California, Davis
Robert C. Green, MD, MPH	Boston University
Andrew J. Saykin, PsyD	Indiana University
Kwangsik Nho, PhD	Indiana University
Richard J. Perrin, MD, PhD	Washington University St. Louis
Duygu Tosun, PhD	University of California, San Francisco

ADNI 4 Private Partner Scientific Board (PPSB) Convened by Alzheimer's Association

Pallavi Sachdev, PhD	Eisai (Chair, 2023-2024)

Data and Publication Committee (DPC)

Robert C. Green, MD, MPH	Harvard University (Chair)
Erin Drake	Harvard University

Resource Allocation Review Committee

Tom Montine, MD, PhD	University of Washington (Chair)
Cat Conti, BA	Northern California Institute for Research and Education

Administrative Core Leaders and Key Personnel

Michael W. Weiner, MD	University of California, San Francisco
Rachel Nosheny, PhD	University of California, San Francisco
Diana Truran Sacrey	Northern California Institute for Research and Education
Juliet Fockler	University of California, San Francisco
Melanie J. Miller, PhD	Northern California Institute for Research and Education
Catherine (Cat) Conti	Northern California Institute for Research and Education
Winnie Kwang, MA	University of California, San Francisco
Chengshi Jin, PhD	University of California, San Francisco
Adam Diaz, MS	Northern California Institute for Research and Education
Miriam Ashford, PhD	Northern California Institute for Research and Education
Derek Flenniken	Northern California Institute for Research and Education
Adrienne Kormos	Northern California Institute for Research and Education

Clinical Core Leaders and Key Personnel

Ronald Petersen, MD, PhD	Mayo Clinic, Rochester (Core PI)
Paul Aisen, MD	University of Southern California (Core PI)
Michael Rafii, MD, PhD	University of Southern California
Rema Raman, PhD	University of Southern California
Gustavo Jimenez, MBS	University of Southern California
Michael Donohue, PhD	University of Southern California
Jennifer Salazar, MBS	University of Southern California
Andrea Fidell, MPH	University of Southern California
Virginia Boatwright, BS	University of Southern California
Justin Robison, MS	University of Southern California
Caileigh Zimmerman, MS	University of Southern California
Yuliana Cabrera, BS	University of Southern California

Sarah Walter, MSc	University of Southern California
Taylor Clanton, MPH	University of Southern California
Elizabeth Shaffer, BS	University of Southern California
Caitlin Webb, BA	University of Southern California
Lindsey Hergesheimer, BS	University of Southern California
Stephanie Smith, BS	University of Southern California
Sheila Ogwang, MPH	University of Southern California
Olusegun Adegoke, MSc	University of Southern California
Payam Mahboubi, MPH	University of Southern California
Jeremy Pizzola, BA	University of Southern California
Cecily Jenkins, PhD	University of Southern California

Biostatistics Core Leaders and Key Personnel

Laurel Beckett, PhD	University of California, Davis (Core PI)
Danielle Harvey, PhD	University of California, Davis (Core PI)
Michael Donohue, PhD	University of Southern California
Naomi Saito, MS	University of California, Davis
Adam Diaz, MS	Northern California Institute for Research and Education
Kedir Adem Hussen, MS	University of Southern California

Engagement Core Leaders and Key Personnel

Ozioma Okonkwo, PhD	University of Wisconsin (Core-PI)
Monica Rivera-Mindt, PhD	Fordham University; Mt. Sinai (Core-PI)
Hannatu Amaza	University of Wisconsin
Mai Seng Thao	University of Wisconsin
Shaniya Parkins	Mt. Sinai
Omobolanle Ayo, MBChB, MPH	Mt. Sinai
Matt Glittenberg	University of Wisconsin
Isabella Hoang	University of Wisconsin
Kaori Kubo Germano, PhD	Fordham University
Joe Strong, PhD	University of Wisconsin
Trinity Weisensel	University of Wisconsin
Fabiola Magana	University of Wisconsin
Lisa Thomas	University of Wisconsin
Vanessa Guzman, PhD	Mt. Sinai
Adeyinka Ajayi, MBBS, MPH	Mt. Sinai
Joseph Di Benedetto, LMSW	Mt. Sinai
Sandra Talavera, MSW	Fordham University

MRI Core Leaders and Key Personnel

Clifford R. Jack, Jr., MD	Mayo Clinic, Rochester (Core PI)

Joel Felmlee, PhD	Mayo Clinic, Rochester
Nick C. Fox, MD	University College London
Paul Thompson, PhD	UCLA School of Medicine
Charles DeCarli, MD	University of California, Davis
Arvin Forghanian-Arani, PhD	Mayo Clinic, Rochester
Bret Borowski, RTR	Mayo Clinic, Rochester
Calvin Reyes	Mayo Clinic, Rochester
Caitie Hedberg	Mayo Clinic, Rochester
Chad Ward	Mayo Clinic, Rochester
Christopher Schwarz, PhD	Mayo Clinic, Rochester
Denise Reyes	Mayo Clinic, Rochester
Jeff Gunter, PhD	Mayo Clinic, Rochester
John Moore-Weiss, PhD	Mayo Clinic, Rochester
Kejal Kantarci, MD	Mayo Clinic, Rochester
Leonard Matoush	Mayo Clinic, Rochester
Matthew Senjem, MS	Mayo Clinic, Rochester
Prashanthi Vemuri, PhD	Mayo Clinic, Rochester
Robert Reid, PhD	Mayo Clinic, Rochester
Ian Malone, PhD	University College London
Sophia I. Thomopoulos, BS	University of Southern California School of Medicine
Talia M. Nir, PhD	University of Southern California School of Medicine
Neda Jahanshad, PhD	University of Southern California School of Medicine
Alexander Knaack, MS	University of California, Davis
Evan Fletcher, PhD	University of California, Davis
Danielle Harvey, PhD	University of California, Davis
Duygu Tosun-Turgut, PhD	University of California, San Francisco
Stephanie Rossi Chen, BA.	Northern California Institute for Research and Education
Mark Choe, BS	Northern California Institute for Research and Education
Karen Crawford	University of Southern California School of Medicine
Paul A. Yushkevich, PhD	University of Pennsylvania
Sandhitsu Das, PhD	University of Pennsylvania

PET Core Leaders and Key Personnel

William Jagust, MD	University of California, Berkeley (Core PI)
Susan Landau, PhD	University of California, Berkeley (Core PI)
Robert A. Koeppe, PhD	University of Michigan
Gil Rabinovici, MD	University of California San Francisco
Victor Villemagne, MD	University of Pittsburgh
Brian LoPresti, MSNE	University of Pittsburgh

Neuropathology Core Leaders and Key Personnel

Richard J. Perrin, MD, PhD	Washington University St. Louis (Core PI)
John Morris, MD	Washington University St. Louis
Erin Franklin, MS	Washington University St. Louis

Haley Bernhardt, BA, R. EEG T.	Washington University St. Louis
Nigel J. Cairns, PhD, MRCPath	Washington University St. Louis
Lisa Taylor-Reinwald, BA, HTL (ASCP)	Washington University St. Louis

Biomarkers Core Leader and Key Personnel

Leslie Shaw, PhD	UPenn School of Medicine (Core PI)
Edward B. Lee, MD, PhD	University of Pennsylvania (Core PI)
Virginia M.Y. Lee, PhD, MBA	UPenn School of Medicine
Magdalena Korecka, PhD	UPenn School of Medicine
Magdalena Brylska, MS	UPenn School of Medicine
Yang Wan, MS	UPenn School of Medicine
J.Q. Trojanowki, MD, PhD*	UPenn School of Medicine (*former Core PI, deceased)

Informatics Core Leader and Key Personnel

Arthur W. Toga, PhD	University of Southern California (Core PI)
Karen Crawford, MLIS	University of Southern California
Scott Neu, PhD	University of Southern California

Genetics Core Leader and Key Personnel

Andrew J. Saykin, PsyD	Indiana University School of Medicine (Core PI)
Kwangsik Nho, PhD	Indiana University School of Medicine (Core PI)
Tatiana M. Foroud, PhD	Indiana University School of Medicine (Dir. NCRAD)
Taeho Jo, PhD	Indiana University School of Medicine
Shannon L. Risacher, PhD	Indiana University School of Medicine
Hannah Craft, MPH	Indiana University School of Medicine
Liana G. Apostolova, MD	Indiana University School of Medicine
Kelly Nudelman, PhD	NCRAD/Indiana University School of Medicine
Kelley Faber, MS, CCRC	NCRAD/Indiana University School of Medicine
Zoë Potter, BA, CCRP	NCRAD/Indiana University School of Medicine
Kaci Lacy, MPH, CCRP	NCRAD/Indiana University School of Medicine
Rima Kaddurah-Daouk, PhD	Duke University/AD Metabolomics Consortium
Li Shen, PhD	University of Pennsylvania

ADNI4 Amyloid PET Visual Read Team

David Soleimani-Meigooni, MD	University of California, San Francisco
Renaud La Joie, PhD	University of California, San Francisco
Konstantinos Chiotis, MD, PhD	University of California, San Francisco
Maison Abu Raya, MD	University of California, San Francisco
Agathe Vrillon, MD, PhD	University of California, San Francisco
Charles Windon, MD	University of California, San Francisco
Julien Lagarde, MD, PhD	University of California, San Francisco
Zoe Lin	University of California, San Francisco
Aidyn Rose Hills	University of California, San Francisco

ADNI4 Amyloid Disclosure Team

Jason Karlawish, MD	University of Pennsylvania
Claire Erickson, PhD	University of Pennsylvania
Joshua Grill PhD	University of California, Irvine
Emily Largent PhD	University of Pennsylvania
Kristin Harkins MPH	University of Pennsylvania

Early Project Development

Michael W. Weiner, MD	UCSF/NCIRE
Leon Thal, MD – Past Investigator	
Zaven Kachaturian, PhD	Khachaturian, Radebaugh & Associates (KRA), Inc
Richard Frank, MD, PhD	General Electric
Peter J. Snyder, PhD	University of Connecticut

Alzheimer's Association's Ronald and Nancy Reagan's Research Institute

NIA

Neil Buckholtz, PhD	National Institute on Aging
John K. Hsiao, MD	National Institute on Aging
Laurie Ryan, PhD	National Institute on Aging
Susan Molchan, PhD	National Institute on Aging/National Institutes of Health

ADNI External Scientific Advisory Board (SAB)

Zaven Khachaturian, PhD	Prevent Alzheimer's Disease 2020 (Chair)
Maria Carrillo, PhD	Alzheimer's Association
William Potter, MD	National Institute of Mental Health
Lisa Barnes, PhD	Rush University
Marie Bernard, MD	NIA
Hector González	University of California, San Diego
Carole Ho	Denali Therapeutics
John K. Hsiao, MD	NIH
Jonathan Jackson, PhD	Massachusetts General Hospital
Eliezer Masliah, MD	NIA
Donna Masterman, MD	Biogen
Ozioma Okonkwo, PhD	University of Wisconsin, Madison
Richard Perrin, MD, PhD	Washington University St. Louis
Laurie Ryan, PhD	NIA
Nina Silverberg, PhD	NIA

Part B: Investigators By Site

Oregon Health and Science University:

Lisa Silbert, MD
Jeffrey Kaye, MD
Sylvia White (Salazar), ND
Aimee Pierce, MD
Amy Thomas, BSN, RN
Tera Clay
Daniel Schwartz, BA
Gillian Devereux, RN, MPH
Janet "Janae" Taylor
Jennifer Ryan, ND, MS
Mike Nguyen
Madison DeCapo, BS
Yanan Shang, MD

University of Southern California:
Lon Schneider, MD
Cynthia Munoz, MA
Diana Ferman, PA
Carlota Conant, BS
Katherin Martin
Kristin Oleary
Sonia Pawluczyk, MD
Elizabeth Trejo
Karen Dagerman
Liberty Teodoro, RN
Mauricio Becerra
Madiha Fairooz, BS
Sonia Garrison, MSsc
Julia Boudreau, MS
Yair Avila, BA

University of California--San Diego:
James Brewer, MD, PhD
Aaron Jacobson
Antonio Gama
Chi Kim
Emily Little, MPH
Jennifer Frascino
Nichol Ferng
Socorro Trujillo, MPH

University of Michigan:
Judith Heidebrink, MD
Robert Koeppe, PhD
Steven MacDonald, MD

Dariya Malyarenko, Ph.D.
Jaimie Ziolkowski, MA, BS, TLLP
James O'Connor, MS, RT (R)(MR)
Nicole Robert
Suzan Lowe
Virginia Rogers

Mayo Clinic, Rochester:
Ronald Petersen, MD, Ph.D.
Barbara Hackenmiller
Bradley Boeve, MD
Colleen Albers, RN
Connie Kreuger
David Jones, MD
David Knopman, MD
Hugo Botha, MB, Ch.B.
Jessica Magnuson
Jonathan Graff-Radford, MD
Kerry Crawley, BSW, CCRP
Michael Schumacher, CNMT
Sanna McKinzie, MS
Steven Smith, MS
Tascha Helland, BS
Val Lowe, MD
Vijay Ramanan, MD, PhD

Baylor College of Medicine:
Valory Pavlik, PhD
Jacob Faircloth, BS
Jeffrey Bishop, PA
Jessica Nath
Maria Chaudhary, MAP
Maria Kataki, PhD, MD
Melissa Yu, MD, FAAN
Nathiel Pacini, MA
Randall Barker
Regan Brooks, BA
Ruchi Aggarwal, MD

Columbia University Medical Center:
Lawrence Honig, MD, Ph.D.
Yaakov Stern, PhD

Akiva Mintz, MD
Jonathan Cordona, ARRT
Michelle Hernandez

Washington University, St. Louis:
Justin Long, MD
Abbey Arnold, NP
Alex Groves
Anna Middleton, RN
Blake Vogler
Cierra McCurry
Connie Mayo, RN
Cyrus Raji, MD, PhD
Fatima S. Amtashar, BS
Heather Klemp, MSW
Heather Nicole Elmore, RN, MSN, ANP-BC, CCRP
James Ruszkiewicz, CNMT
Jasmina Kusuran
Jasmine Stewart
Jennifer Horenkamp, RN, BSN
Julia Greeson, MS
Kara Wever, MA
Katie Vo, MD
Kelly Larkin, RN
Lesley Rao, MD
Lisa Schoolcraft, BFA
Lora Gallagher
Madeline Paczynski, BS, PA-C
Maureen McMillan
Michael Holt, MSW
Nicole Gagliano, BS, RT
Rachel Henson, MS
Renee LaBarge
Robert Swarm, MD
Sarah Munie, BSN, RN
Serena Cepeda, BS
Stacey Winterton, BSN, RN
Stephen Hegedus
TaNisha Wilson
Tanya Harte, FNP-BC
Zach Bonacorsi

University of Alabama Birmingham:
David Geldmacher, MD

Amber Watkins, RN
Brandi Barger, BSRT
Bryan Smelser, MD
Charna Bates, MA
Cynthia Stover, PENDING
Emily McKinley,
Gregory Ikner, MA
Haley Hendrix,
Harold Matthew Cooper, MSN, CRNP, NPC
Jennifer Mahaffey,
Lindsey Booth Robbins, MSN, CRNP, PNP-C
Loren Brown Ashley, RN, BSN
Marissa Natelson-Love, MD
Princess Carter, RN
Veronika Solomon,

Mount Sinai School of Medicine:
Hillel Grossman, MD
Alexandra Groome, BA
Allison Ardolino, MA
Anthony Kaplan, ARRT, CNMT
Faye Sheppard, BS
Genesis Burgos-Rivera, BA
Gina Garcia-Camilo, MD
Joanne Lim, MA
Judith Neugroschl, MD
Kimberly Jackson, BS
Kirsten Evans, BS
Laili Soleimani, MD
Mary Sano, Ph.D.
Nasrin Ghesani, MD
Sarah Binder, BS
Xiomara Mendoza Apuango, BS

Rush University Medical Center:
Ajay Sood, MD, PhD
Amelia Troutman, MA
Kimberly Blanchard, APRN, DNP, NP-C
Arlene Richards,
Grace Nelson, BA
Kirsten Hendrickson, RN, MSN
Erin Yurko,
Jamie Plenge, BS
Victoria Rufo, MS
Raj Shah, MD

Wein Center:
Ranjan Duara, MD
Brendan Lynch, CRT
Cesar Chirinos, PsyD
Christine Dittrich, CRT
Debbie Campbell
Diego Mejia, CRT
Gilberto Perez, CRT
Helena Colvee, BS
Joanna Gonzalez, PsyD
Josalen Gondrez, MS
Joshua Knaack
Mara Acevedo
Maria Cereijo, APRN
Maria Greig-Custo, MD
Michelle Villar, BS
Morris Wishnia
Sheryl Detling
Warren Barker, MS

Johns Hopkins University:
Marilyn Albert, Ph.D.
Abhay Moghekar
Barbara Rodzon
Corey Demsky
Gregory Pontone, MD
Jim Pekar
Leonie Farrington, CNRN
Martin Pomper
Nicole Johnson
Tolulope Alo

New York University:
Martin Sadowski, MD, PhD
Anaztasia Ulysse, BA
Arjun Masurkar
Brittany Marti
David Mossa, R.T
Emilie Geesey
Emily Petrocca, NP
Evan Schulze, PhD
Jennifer Wong
Joseph Boonsiri
Sunnie Kenowsky, DVM

Tatianne Martinez, NP
Veronica Briglall

Duke University Medical Center:
P. Murali Doraiswamy, MD, MBBS
Adaora Nwosu
Alisa Adhikari, BS
Cammie Hellegers, MA
Jeffrey Petrella
Olga James, MD
Terence Wong
Thomas Hawk

University of Pennsylvania:
Sanjeev Vaishnavi, MD, PhD
Hannah McCoubrey, BA
Ilya Nasrallah, MD, PhD
Rachel Rovere, BA
Jeffrey Maneval, MD
Elizabeth Robinson, MA
Francisco Rivera, MS
Jade Uffelman, BS
Martha Combs, BS, MS
Patricia O'Donnell
Sara Manning, MD

University of Kentucky:
Richard King, MD
Alayne Nieto, BSN, RN
Amanda Glueck, PhD
Anjana Mandal
Audrie Swain
Bethanie Gamble, PhD, RN
Beverly Meacham, RT(R) (MR)
Denece Forenback, RN
Dorothy Ross, CCRP
Elizabeth Cheatham
Ellen Hartman
Gary Cornell
Jordan Harp, PhD
Laura Ashe
Laura Goins
Linda Watts, RN
Morgan Yazell
Prabin Mandal

Regan Buckler, BSN, RN
Sylvia Vincent
Triana Rudd

University of Pittsburgh:
Oscar Lopez, MD
Ann Arlene Malia
Caitlin Chiado, CRNP
Cary Zik
James Ruszkiewicz, CNMT
Kathleen Savage
Linda Fenice
MaryAnn Oakley, MA
Paige C Tacey, M.Ed.
Sarah Berman, MD, PhD
Sarah Bowser, CRNP
Stephen Hegedus
Xanthia Saganis

University of Rochester Medical Center:
Anton Porsteinsson, MD
Abigail Mathewson, RN, BSN
Asa Widman, BA
Bridget Holvey, BS
Emily Clark, DO
Esmeralda Morales, MS
Iris Young, PA-C
James Ruszkiewicz, CNMT
Kevin Hopkins, BS, CNMT, LNMT
Kimberly Martin, RN, BSN
Nancy Kowalski, RN, MS
Rebecca Hunt, BS
Roberta Calzavara, PhD
Russell Kurvach, BS, CCRP
Stephen D'Ambrosio, PA-C, MPAS

University of California, Irvine:
Gaby Thai, MD
Beatriz Vides, RN, MSN
Brigit Lieb, ARRT/CRT
Catherine McAdams-Ortiz, MSN, RN, A/GNP
Cyndy Toso
Ivan Mares, BS
Kathryn Moorlach

Luter Liu
Maria Corona, PhD
Mary Nguyen, BA
Melanie Tallakson, DNP, FNP-C
Michelle McDonnell, PhD
Milagros Rangel, BS
Neetha Basheer, MD, MBBS
Patricia Place, BA
Romina Romero, PhD
Steven Tam, MD

University of Texas Southwestern Medical School:
Trung Nguyen, MD, PhD
Abey Thomas, ARRT
Alexander (Alex) Frolov, MD
Alka Khera, MD
Amy Browning, BA (Pending)
Brendan Kelley (031), MD
Courtney Dawson, RT(R)
Dana Mathews, MD, Ph.D.
Elaine Most, MS (Pending)
Elizeva (Ellie) Phillips, CNMT
Lynn Nguyen
Maribel Nunez
Matalin Miller, MS
Matthew R. Jones, MA
Natalie Martinez, MSN, RN, FNP-BC
Rebecca Logan, PA-C
Roderick McColl
Sari Pham
Tiffani Fox, MBA, MS
Tracey Moore, BA

Emory University:
Allan Levey, MD, PhD
Abby Brown, NP
Andrea Kippels, NP
Ashton Ellison, BSPH, ABA
Casie Lyons
Chadwick Hales, MD, PhD
Cindy Parry, BFA
Courtney Williams
Elizabeth McCorkle, BS
Guy Harris, BA

Heather Rose, BSN
Inara Jooma, BS
Jahmila Al-Amin, MS, BS
James Lah, MD, PhD
James Webster, BS
Jessica Swiniarski, MPH, BS
Latasha Chapman, BS
Laura Donnelly, MPH
Lauren Mariotti
Mary Locke, BS
Phyllis Vaughn, BSN
Rachael Penn, BSN, RN
Sallie Carpentier, RN, BSN
Samira Yeboah, BMSc, R.T.(R) (MR)
Sarah Basadre, BMSc, ARRT(R)(MR)
Sarah Malakauskas, MS
Stefka Lyron, NP
Tara Villinger, NP
Terra Burney

University of Kansas, Medical Center:
Jeffrey Burns, MD, MS
Ala Abusalim, PA-C
Alexandra Dahlgren, BS
Alexandria Montero, RN
Anne Arthur, BSN, MS, ANP-BC
Heather Dooly, BS
Katelynn Kreszyn, APRN
Katherine Berner, BS
Lindsey Gillen, APRN
Maria Scanlan, BA
Mercedes Madison, BS
Nicole Mathis
Phyllis Switzer
Ryan Townley, MD
Samantha Fikru, APRN, MSN, FNP-C
Samantha Sullivan, MSW
Ella Wright, BS

University of California, Los Angeles:
Maryam Beigi, MD
Anthony Daley
Ashley Ko
Brittney Luong
Glen Nyborg

Jessica Morales
Kelly Durbin, PhD
Lauren Garcia
Leila Parand
Lorena Macias
Lorena Monserratt, PhD
Maya Farchi
Pauline Wu, DO
Robert Hernandez
Thao Rodriguez, NP

Mayo Clinic, Jacksonville:
Neill Graff-Radford, MD, MBBCH, FRCP
A'llana Marolt, BS
Anton Thomas, BS
Deborah Aloszka
Ercilia Moncayo, BS
Erin Westerhold, RT
Gregory Day, MD
Kandise Chrestensen, BS
Mary Imhansiemhonehi, BS
Sanna McKinzie, MS
Sochenda Stephens, CCRP
Sylvia Grant, CCRC

Indiana University:
Jared Brosch, MD
Amy Perkins, CCRP
Aubree Saunders, BS
Debra Silberberg Kovac, BS
Heather Polson, CNMT
Isabell Mwaura, BS
Kassandra Mejia, BS
Katherine Britt, BS
Kathy King, RN
Kayla Nichols, BS
Kayley Lawrence, BA
Lisa Rankin, BSW
Martin Farlow, MD
Patricia Wiesenauer, MS
Robert Bryant, BS
Scott Herring, RN
Sheryl Lynch, RN
Skylar Wilson
Traci Day

William Korst

Yale University School of Medicine:
Christopher van Dyck, MD
Adam Mecca, MD, PhD
Alyssa Miller, BS
Amanda Brennan, LMSE, MSW
Amber Khan, MD
Audrey Ruan
Carol Gunnoud, AS
Chelsea Mendonca, MD
Danielle Raynes-Goldfinger, BS
Elaheh Salardini, MD
Elisa Hidalgo, MS, CNMT, EMT, RT (CT)
Emma Cooper, BA
Erawadi Singh, DO
Erin Murphy, BS
Jeanine May, APRN, MSN, MHP, CCRP
Jesse Stanhope, BS
Jessica Lam, BSE
Julia Waszak, BS
Kimberly Nelsen, BA
Kimberly Sacaza, BS
Mayer Joshua Hasbani, MD
Meghan Donahue, BA
Ming-Kai Chen, MD, PhD
Nicole Barcelos, MS, MA
Paul Eigenberger, MD
Robin Bonomi, MD
Ryan O'Dell, MD, PhD
Sarah Jefferson, MD
Siddharth Khasnavis, MD
Stephen Smilowitz, MD
Susan DeStefano, APRN, MSN
Susan Good, APRN
Terry Camarro, RT, RN, MRI, APRT
Vanessa Clayton, BS
Yanis Cavrel, BA
YuQuan "Oliver" Lu

McGill University, Montreal-Jewish General Hospital:
Howard Chertkow, MD
Howard Bergman, MD
Chris Hosein, M.Ed

Sunnybrook Health Sciences, Ontario:
Sandra Black, MD
Anish Kapadia, MD
Aparna Bhan
Benjamin Lam, MD, FRCP(c)
Christopher Scott, BSc
Gillian Gabriel, MA
Jennifer Bray, BA, BSW, MSW
Ljubica Zotovic, MD
Maria Samira Gutierrez
Mario Masellis
Marjan Farshadi, MD
Maurylette Gui, Psych BSc
Meghan Mitchell, BSc
Rebecca Taylor
Ruby Endre, M.R.T
Zhala Taghi-Zada

University of British Columbia Clinic for AD & Related Disorders
Robin Hsiung, MD
Carolyn English
Ellen Kim, BA
Eugene Yau
Haley Tong
Laura Barlow, RTR/RTMR
Lauren Jennings
Michele Assaly
Paula Nunes, PhD
Tahlee Marian

Cognitive Neurology St. Joseph's Ontario:
Andrew Kertesz, MD
John Rogers, MD
Dick Trost, PhD

Cleveland Clinic Lou Ruvo Center for Brain Health
Dylan Wint, MD
Charles Bernick, MD
Donna Munic, PhD

Northwestern University:
Ian Grant, MD
Aaliyah Korkoyah, BS
Ali Raja
Allison Lapins, MD

Caila Ryan, MS
Jelena Pejic
Kailey Basham, BS
Leena Lukose, BS
Loreece Haddad, MS
Lucas Quinlan, BS, MLS (ASCP)
Nathaniel Houghtaling

Premiere Research Inst (Palm Beach Neurology):
Carl Sadowsky MD
Walter Martinez MD
Teresa Villena MD

Georgetown University Medical Center:
Brigid Reynolds, NP
Angelica Forero, MS
Carolyn Ward, MSPH
Emma Brennan, BS
Esteban Figueroa
Giuseppe Esposito, MD
Jessica Mallory
Kathleen Johnson, RN, NP
Kathryn Turner, BSN
Katie Seidenberg
Kelly McCann, BA
Margaret Bassett, NP
Melanie Chadwick, NP
Raymond Scott Turner, MD, PhD
Robin Bean, RT
Saurabh Sharma, MD

Brigham and Women's Hospital:
Gad Marshall, MD
Aferdita Haviari, BA
Alison Pietras, PA-C, ACP
Bradley Wallace, BS
Catherine Munro, PhD
Gladiliz Rivera-Delpin, MA
Hadley Hustead, BS
Isabella Levesque
Jennifer Ramirez, BA
Karen Nolan, BS, RT (MR)
Kirsten Glennon, RN, CNRN
Mariana Palou, BA
Michael Erkkinen, MD

Nicole DaSilva
Pamela Friedman, Psy. D
Regina M. Silver, RN
Ricardo Salazar, MD
Roxxanne Polleys, AA
Scott McGinnis (094), MD
Seth Gale, MD
Tia Hall, BS
Tuan Luu

Stanford University:
Steven Chao, MD
Emmeline Lin, BS
Jaila Coleman, BA
Kevin Epperson, RT(R)(MR)
Minal Vasanawala

Banner Sun Health Research Institute
Alireza Atri, MD, PhD
Amy Rangel
Brittani Evans
Candy Monarrez
Carol Cline, LMSW
Carolyn Liebsack, RN, BSN, CCRC
Daniel Bandy
Danielle Goldfarb, MD
Debbie Intorcia
Jennifer Olgin
Kelly Clark
Kelsey King, CCRP
Kylee York
Marina Reade, RN, FNP-C
Michael Callan
Michael Glass
Michaela Johnson, G-ACNP, BC
Michele Gutierrez
Molly Goddard
Nadira Trncic, MD, PhD
Parichita Choudhury, MD
Priscilla Reyes
Serena Lowery
Shaundra Hall
Sonia Olgin
Stephanie de Santiago, RN, NP

Boston University:
Michael Alosco, PhD
Alyssa Ton, BS
Amanda Jimenez, MS, EMT-B, CPT
Andrew Ellison, MR Technologist
Anh Tran, RN
Brandon Anderson, RT(N), CNMT
Della Carter, MS
Donna Veronelli, RTN, CNMT
Steven Lenio, MD
Eric Steinberg, RN, MSN, CNP
Jesse Mez, MD, MS
Jason Weller, MD
Jennifer Johns, RN
Jesse Mez, MD, MS
Jessica Harkins, CNMT
Alexa Puleio, MS
Ina Hoti, BS
Jane Mwicigi, MBChB., MPH
Alexa Puleio, MS
Michael Alosco, PhD
Olivia Schultz, BA
Mona Lauture, RN
Eric Steinberg
Ridiane Denis, RN
Ronald Killiany, PhD
Sarab Singh, CNMT
Steven Lenio, MD
Wendy Qiu, MD, PhD
Ycar Devis, MPH

Howard University:
Thomas Obisesan, MD, MPH
Andrew Stone, MS
Debra Ordor, RN, BSN
Ifreke Udodong, CRNP
Immaculata Okonkwo, DNP, MSN, APRN, FNP-BC
Javed Khan, MD
Jillian Turner, BS, MS
Kyliah Hughes, BS, RMA
Oshoze Kadiri, MPH

Case Western Reserve University:
Charles Duffy, MD, PhD

Ariana Moss
Katherine Stapleton, LPN
Maria Toth (fmr Gross), RN
Marianne Sanders, BSN, RN
Martin Ayres
Melissa Hamski
Parianne Fatica, CCRC
Paula Ogrocki, PhD
Sarah Ash
Stacy Pot

University of California, Davis Sacramento :
Doris Chen, MD
Andres Soto
Costin Tanase, PhD
David Bissig, MD, PhD
Hafsanoor Vanya, BA
Heather Russell (116), CNMT
Hitesh Patel, CNMT
Hongzheng Zhang, CCRP
Kelly Wallace, CCRP
Kristi Ayers, BS
Maria Gallegos, BS
Martha Forloines, PhD
Meghan Sinn
Queennie Majorie S Kahulugan, CCRC
Richard Isip, RT (R)(N)(CT)
Sandra Calderon, MS, RN, FMP-C
Talia Hamm, BA, CCRP

Parkwood Hospital:
Michael Borrie, MD
T-Y Lee, PhD
Dr Rob Bartha, PhD

University of Wisconsin:
Sterling Johnson, PhD
Sanjay Asthana, MD
Cynthia M. Carlsson, MD

Banner Alzheimer's Institute:
Allison Perrin, MD
Pierre Tariot, MD
Adam Fleisher, MD
Stephanie Reeder, BA

Dent Neurologic Institute
Horacio Capote, MD
Allison Emborsky
Anna Mattle, PharmD, MS
Bela Ajtai, MD
Benjamin Wagner, PA-C
Bennett Myers
Daryn Slazyk
Delaney Fragale, PA-C
Erin Fransen, PA
Heather Macnamara
Jonathan Falletta, PA-C
Joseph Hirtreiter, RN
Laszlo Mechtler, MD
Megan King
Michael Asbach, RPA-C
Michelle Rainka, Pharm. D., CCRP
Richard Zawislak, NP
Scott Wisniewski
Stephanie O'Malley, PA-C
Tatiana Jimenez-Knight
Todd Peehler
Traci Aladeen, PharmD
Vernice Bates
Violet Wenner
Wisam Elmalik, MD

Ohio State University:
Douglas W. Scharre, MD
Arun Ramamurthy, MD
Soumya Bouchachi, MD
Maria Kataki, MD, PhD - Past Investigator
Rawan Tarawneh, MD - Past Investigator
Brendan Kelley, MD - Past Investigator

Albany Medical College:
Dzintra Celmins, MD
Alicia Leader
Chris Figueroa
Heather Bauerle, NP
Katlynn Patterson
Michael Reposa
Steven Presto
Tuba Ahmed

Wendy Stewart

Hartford Hosp, Olin Neuropsychiatry Research Center:
Godfrey D. Pearlson MD
Karen Blank, MD
Karen Anderson, RN

Dartmouth-Hitchcock Medical Center:
Robert B. Santulli, MD
Eben S. Schwartz, PhD

Wake Forest University Health Sciences:
Jeff Williamson, MD, MHS, FACP
Alicia Jessup, RN
Andrea Williams
Crystal Duncan
Abigail O'Connell, APRN, FNP-C
Karen Gagnon
Ezequiel Zamora
James Bateman
Freda Crawford, CNMT
Deb Thompson
Eboni Walker
Jennifer Rowell
Mikell White, MHA
Phillip "Hunter" Ledford
Sarah Bohlman, MSL
Susan Henkle, RN
Joseph Bottoms, CNMT
Lena Moretz, RT(R) CT (MR)
Bevan Hoover, BS
Michael Shannon
Samantha Rogers, PA-C
Wendy Baker
William Harrison, MD

Rhode Island Hospital:
Chuang-Kuo Wu, MD
Alexis DeMarco, BS
Ava Stipanovich, BS, ScM
Daniel Arcuri, CNMT, RT(N)(CT)
Jan Clark, RN, BSN, CCRC, CSNT
Jennifer Davis, PhD
Kerstin Doyon, RN, BSN

Marie Amoyaw, BA
Mauro Veras Acosta, PENDING, BS
Ronald Bailey, RT-R, CNMT
Scott Warren, MD
Terry Fogerty
Victoria Sanborn, PhD

Butler Hospital
Meghan Riddle, MD
Stephen Salloway, MD, MS
Paul Malloy, PhD
Stephen Correia, PhD

University of California San Francisco
Charles Windon, MD
Morgan Blackburn
Howard J. Rosen, MD
Bruce L. Miller, MD

University of South Florida, Byrd Institute
Amanda Smith, MD
Ijeoma Mba, MBA, MPH
Jenny Echevarria
Juris Janavs

University of Chicago
Emily Roglaski, PhD
Meagan Yong
Rebecca Devine

Eastern Virginia Medical School
Hamid Okhravi, MD

Charter Health Research Services
Edgardo Rivera, MD
Teresa Kalowsky
Caroline Smith
Christina Rosario

Houston Methodist Neurological Institute
Joseph Masdeu, MD, PhD
Richard Le, PharmD
Maushami Gurung

Barrow Neurological Institute
Marwan Sabbagh, MD
Angelica Garcia
Micah Ellis Slaughter
Nadeen Elayan
Skieff Acothley

Nathan Kline Institute
Nunzio Pomara, MD
Raymundo Hernando
Vita Pomara
Chelsea Reichert

Ralph Johnson Veterans Administration Health Care Services
Olga Brawman-Mintzer, MD
Allison Acree
Arthur Williams
Campbell Long
Rebecca Long

Vanderbilt University Medical Center
Paul Newhouse, MD
Sydni Jenee Hill
Amy Boegel

University of Texas Health, San Antonio
Sudha Seshadri, MD
Amy Saklad
Floyd Jones

Rutgers University
William Hu, MD, PhD
V. Sotelo

Gonzalez & Aswad Health Services
Yaneicy Gonazalez Rojas, MD

Medical University South Carolina
Jacobo Mintzer, MD, MBA
Crystal Flynn Longmire, PhD
Kenneth Spicer, MD, PhD
